# Network or regression-based methods for disease discrimination: a comparison study

**DOI:** 10.1186/s12874-016-0207-2

**Published:** 2016-08-18

**Authors:** Xiaoshuai Zhang, Zhongshang Yuan, Jiadong Ji, Hongkai Li, Fuzhong Xue

**Affiliations:** Department of Epidemiology and Biostatistics, School of Public Health, Shandong University, PO Box 100, Jinan, 250012 China

**Keywords:** Disease discrimination, AUC, Network-based, Regression-based

## Abstract

**Background:**

In stark contrast to network-centric view for complex disease, regression-based methods are preferred in disease prediction, especially for epidemiologists and clinical professionals. It remains a controversy whether the network-based methods have advantageous performance than regression-based methods, and to what extent do they outperform.

**Methods:**

Simulations under different scenarios (the input variables are independent or in network relationship) as well as an application were conducted to assess the prediction performance of four typical methods including Bayesian network, neural network, logistic regression and regression splines.

**Results:**

The simulation results reveal that Bayesian network showed a better performance when the variables were in a network relationship or in a chain structure. For the special wheel network structure, logistic regression had a considerable performance compared to others. Further application on GWAS of leprosy show Bayesian network still outperforms other methods.

**Conclusion:**

Although regression-based methods are still popular and widely used, network-based approaches should be paid more attention, since they capture the complex relationship between variables.

**Electronic supplementary material:**

The online version of this article (doi:10.1186/s12874-016-0207-2) contains supplementary material, which is available to authorized users.

## Background

Recently, an explosion of data has been derived from clinical or epidemiological researches on specific diseases, and the advent of high-throughput technologies also brought an abundance of laboratory data [1–4]. The acquired variables may range from subject general characteristics, history, physical examination results, blood, to a particularly large set of genetic markers. It is desirable to develop efficient data mining strategies to extract more information rather than put the data aside. Diagnostic prediction models are widely applied to guide clinical professionals in their decision making by estimating an individual’s probability of having a specific disease [5–9]. One common sense is, from a network-centric perspective, biological phenomena depend on the interplay of different levels of components [10–12]. For data on network structure, complex relationships (e.g. high collinearity) inevitably exist in large sets of variables, which pose great challenges on conducting statistical analysis properly. Therefore, it is often hard for clinical researchers to determine whether and when to use which exact model to support their decision making.

Regression-based methods, although may be unreasonable to some extent under the network framework, is still a priority in disease diagnosis or discrimination problem [6, 13–15], which is easier to be accepted by clinical researchers due to the interpretability of model parameters and ease of use. However, for regression model, some assumptions needed to be made may limit the use, such as linearity and additivity [16–18]. The performance of the regression model can be affected by the collinearity between the input variables, which is commonly encountered in dataset with complex relationship. Although a logistic regression model can consider the relationship between the covariates by adding interaction terms, the number of possible interactions increases exponentially as the number of input variables increases, resulting in the complex process of specification of interaction and inevitably low power.

To overcome the above problems, numerous machine learning methods have emerged as potential alternatives to logistic regression analysis, such as neural network, random forest, decision trees [5, 19–21]. Neural networks, with few assumptions about the data distribution, can reflect the complex nonlinear relationships between the predictor variables and the outcome by the hidden nodes in the hidden layer. This not only greatly simplifies the modeling work compared to logistic regression model but enables us to model complex forms between variables. If the logistic sigmoid activation function is used, the network without a hidden layer is actually identical to a logistic regression model, and neural networks can be thought as a weighted average of logit functions with the weights themselves estimated [22, 23]. Neural networks do not yet jump out from the scope of regression, which can be viewed as a type of non-parametric regression method.

Motivated by the network perspective, a more formal and visualized representation, usually offered by mathematical graph theory, seems to be more appropriate to describe the biological phenomena. Among these, Bayesian networks provide a systematic method for structuring probabilistic information about a network, which have been receiving considerable attention over the last few decades in a number of research fields [24–26]. Bayesian networks are easily understood since they represent knowledge through a directed acyclic graph (DAG) with nodes and arrows. The network structure can be either generated from data by structural learning or elicited from experts. It could not only avoid statistical assumptions, but also handle the relationship between a larger numbers of predictors with their interactions.

In stark contrast to commonly accepted network-centric perspective view for complex disease, regression-based methods are preferred, especially for epidemiologists and clinical professionals, which usually lead to considerate and easily interpreted results. It remains a controversy whether the network-based methods have advantageous performance than others in discrimination ability, and to what extent do they outperform. In particular, complex diseases often result from multiple genes or molecules interplays within biological pathways or gene regulatory networks. Under such condition, are regression-based methods with correlated genetic markers sufficient to reflect biological reality? To the best of our knowledge, few attempts were conducted to determine in which case network or regression-based methods should be applied. The focus of this paper is, through a series of simulations, to assess how the network-based methods work compared to regression-based methods in prediction performance under different scenarios (the input variables are independent or in network relationship). To achieve this goal, we applied logistic regression, neural network, and Bayesian network on the different datasets.

## Method

### Simulation studies

Simulation studies were conducted to evaluate the performance of the logistic regression, neural network, and Bayesian network. The area under the receiver-operating characteristic curve (AUC) which is normally employed to measure discrimination ability [27],and Brier score was used to compare the accuracy of the three methods. Additional techniques (e.g. cross-validation (CV), bootstrapping, leverage correction) [28] must be used to alleviate overfitting problem generally encountered in statistics and machine learning. In this paper, the overfitting was corrected using 10-fold cross validation (AUC-CV) to assess the prediction performance of the above three methods. For each simulation, 100 repeats of 10-fold CV were conducted in order to yield sufficient precision.

Under the null hypothesis, the AUC should be around 0.5, meaning that the prediction model is not helpful at all. In order to test whether the prediction methods are stable, we first generated the datasets under the null hypothesis. Network datasets were generated using software Tetrad [29]. For each network, we first generated a directed acyclic graph with a set of binary variables representing the input variables and a binary outcome variable indicating the disease status. Conditional probability table for each variable was defined subsequently. Conditional on the values of its parent variables, there is a defined probability that a variable will take on its possible values. Thus the influence of variables can be reflected by the conditional probability table. Restricting on six nodes including five input variables and one disease outcome, we considered two scenarios of the null hypsthesis: 1) each variable was generated independently; 2) the input variables were network constructed but not associated with the disease. For each scenario, 100,000 individuals were generated to form a hypothetical population from which the samples were randomly selected with different sample sizes (*N* = 30, 50, 100, 200, 500 or 1000). To examine the stability of the three methods, we randomly sampled N individuals respectively for the calculation of the AUC and the average AUC-CV. A total of 1000 simulations were repeated for each sample size.

Under the alternative hypothesis, datasets from different network structures were generated to assess the discriminatory ability as well as the prediction accuracy. We simulated a regular network and two extreme scenarios including chain network and wheel network to evaluate the performance of three different methods. For each data set, similar simulations were accomplished as above to obtain the AUC and the Brier score with different sample size. In particular, more general logistic models were employed to extract the nonlinear effect and interactions between variables for data in regular network. Multivariate regression splines was used to fit the logistic model using *earth* function in R package earth. We used two strategies to consider the interaction between the input variables: 1) the product term was determined by the network structure (i.e. the product term between two variables was added to the model only if there was an edge between the variables). 2) all the pair-wise product terms between the variables were added in the logistic model and selected by stepwise algorithm.

In addition, we might be also interested in how the network methods perform under the special case when the input variables are in completely linear relationship. We generated 100,000 individuals with five independent variables, with each variable following a Binomial distribution. Given the effect of the input variables *β* = (1.5, 1.5, 1.5, 1.5, 1.5), the binary response indicating disease status was generated using logistic regression model.

The performances of Bayesian network and neural network were implemented using the R package bnlearn and the R package neuralnet. For Bayesian network, score-based structure algorithms hill climbing (HC) method (*hc* function) was employed for structure learning and Bayes method for parameter learning (*bn.fit* function). The *neuralnet* function was used to fit the neural network, and the number of hidden nodes in neural network was determined using cross validation.

### Application

The Bayesian network, neural network, logistic regression and regression splines were also applied to a real genotype data for predicting leprosy of Han Chinese with a case control design, which contains 706 cases and 514 controls. The genetically unmatched controls were removed to avoid population stratification. Previous genome-wide association study (GWAS) of leprosy of Han Chinese [30] has identified significant associations between 16 SNPs in seven genes (CCDC122, C13orf31, NOD2, NFSF15, HLA-DR, RIPK2and LRRK2). In this paper, we fitted the three models using the identified 16 SNPs respectively to compare their abilities in predicting Leprosy. The 100 repeats of AUC and Brier score with cross validation were calculated for all the methods.

## Result

**Fig. 1:**
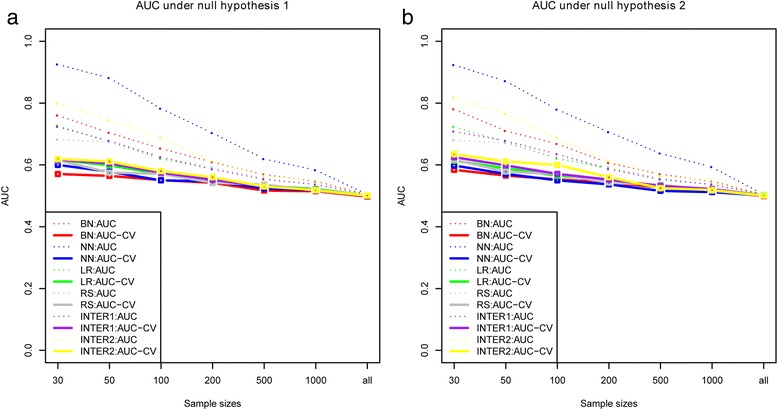
The cross-validation AUC of the Bayesian network, neural network, logistic regression, and regression splines under the null hypothesis. **a** depicts the null hypothesis when each variable including both input and disease was generated independently; **b** shows the null hypothesis when the input variables were network constructed but not associated with the disease

Figure 1 shows the estimated AUC and the average AUC-CV of the Bayesian network, neural network and logistic regression under the null hypothesis mentioned above. It reveals that the AUC-CV of all the methods are close to 0.5 when the sample size is large (more than 500), illustrating the AUC-CV could be a convincing indicator to assess the prediction performance. While AUC is far from 0.5 especially with small sample size and might not be considered in the comparison.

**Fig. 2:**
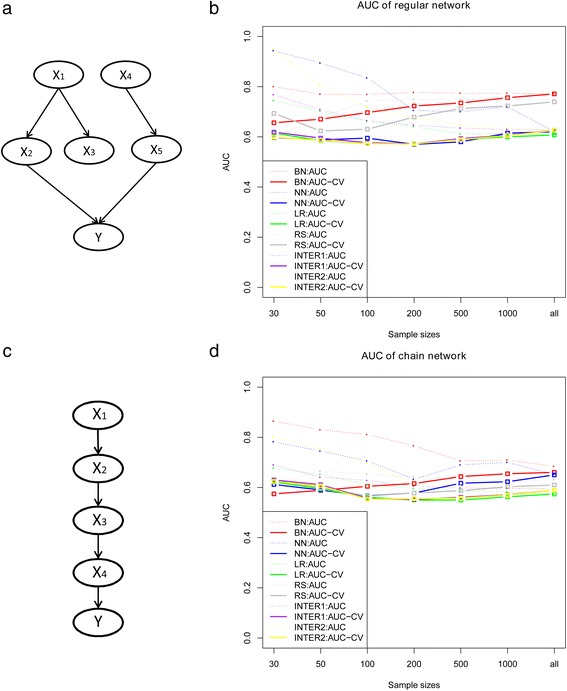
The cross-validation AUC of the methods with regular network structure and chain network structure. **a** depicts the structure of the regular network and **b** shows the cross-validation AUC of Bayesian network, neural network, logistic regression, and regression splines; **c** shows the chain network structure while **d** depicts the cross-validation AUC

Figure 2a shows a simulated disease network, this network data were generated through software Tetrad [29] under the given conditional probabilities. Figure 2b depicts the average AUC-CV slightly increase monotonically by sample size, and they are close to the true value when sample size arrives 1000. The result indicates that Bayesian network outperforms the logistic regression and neural network when such network exists. The logistic regression with interaction terms improved the AUC-CV quite slightly, while regression splines improved the discriminatory ability by capturing the non-linear effect. Table 1 depicts the Brier scores of the methods. The Bayesian network still has the best prediction accuracy, followed by the regression splines. The other four methods have comparably inferior performance.

**Table 1:** Brier score of all the methods for regular network

Method	Brier score with 10-fold CV					
	30	50	100	200	500	1000
Bayesian network	0.236	0.222	0.209	0.201	0.198	0.191
Regression Spline	0.286	0.265	0.235	0.2182	0.210	0.208
Neural network	0.323	0.278	0.248	0.246	0.241	0.233
Logistic Regression	0.317	0.281	0.259	0.250	0.243	0.242
Interaction 1	0.335	0.289	0.263	0.251	0.244	0.241
Interaction 2	0.452	0.351	0.279	0.257	0.246	0.242

Figure 2d shows the performance under different sample sizes given the datasets generated from chain network (Fig. 2c). It seems that the AUC-CV of all methods are not significantly affected by sample size. The Bayesian network has superior performance followed by the neural network, while the regression models work inefficiently that may be partly due to the correlated structure between the input variables. Similar trends can be found for Brier score of the methods.

**Fig. 3:**
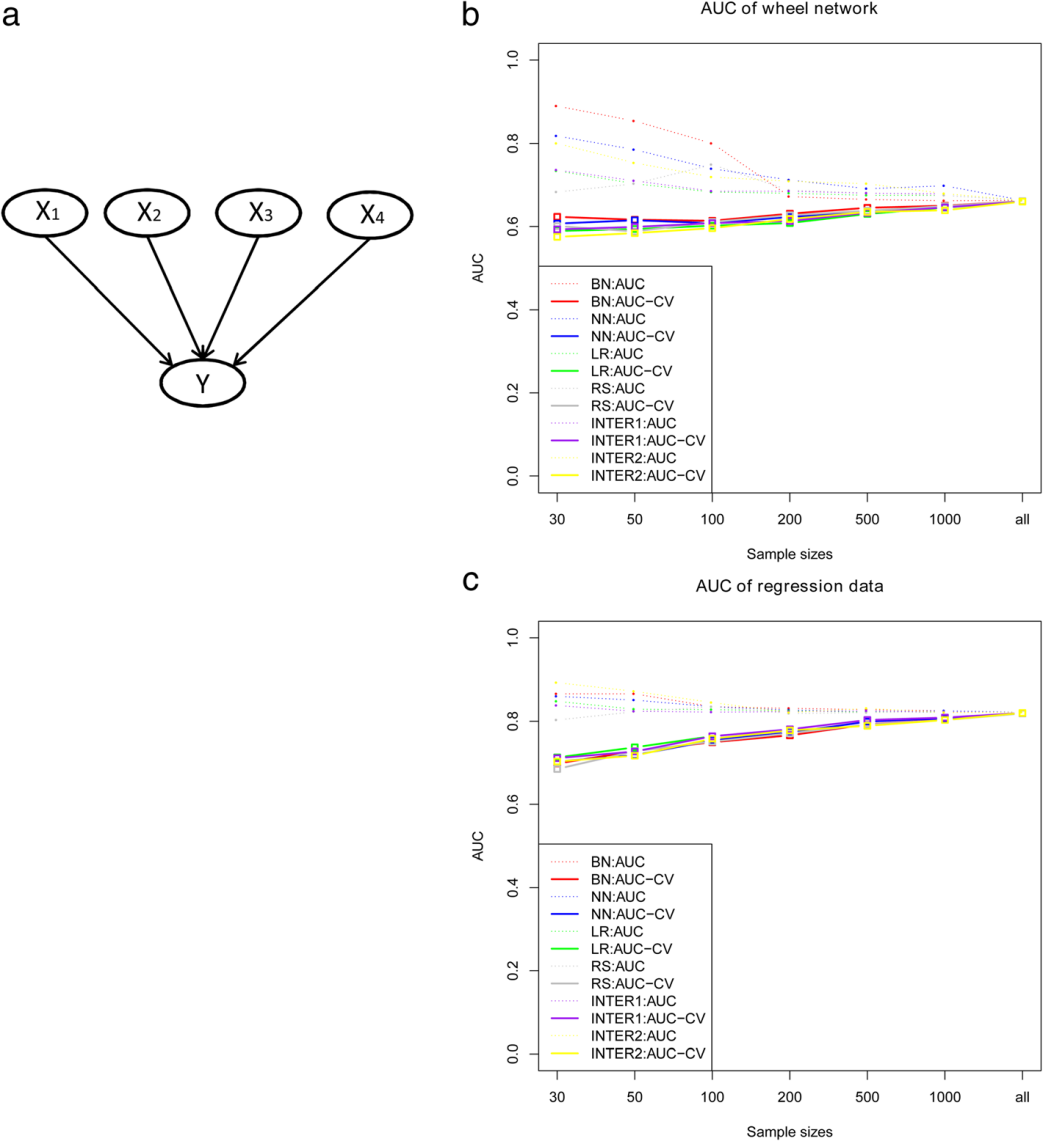
The cross-validation AUC of the methods with wheel network structure and data simulated by logistic model. **a** depicts the structure of the wheel network and **b** shows the cross-validation AUC of Bayesian network, neural network logistic regression, and regression splines; **c** shows the cross-validation AUC for data simulated by logistic model

Given the datasets generated from wheel network shown in Fig. 3a, it depicts the discriminatory ability and accuracy of all these methods are comparable, while the regression models have slightly inferior performance with small sample size. Figure 3c demonstrates that the 10-fold cross-validation AUC of these methods slightly increase monotonically by sample size, while the Brier score decrease monotonically by sample size (please see Additional file 1: Table S3). The prediction ability of the methods are quite close when the independent variables satisfied the linearity.

### Result of application

**Fig. 4:**
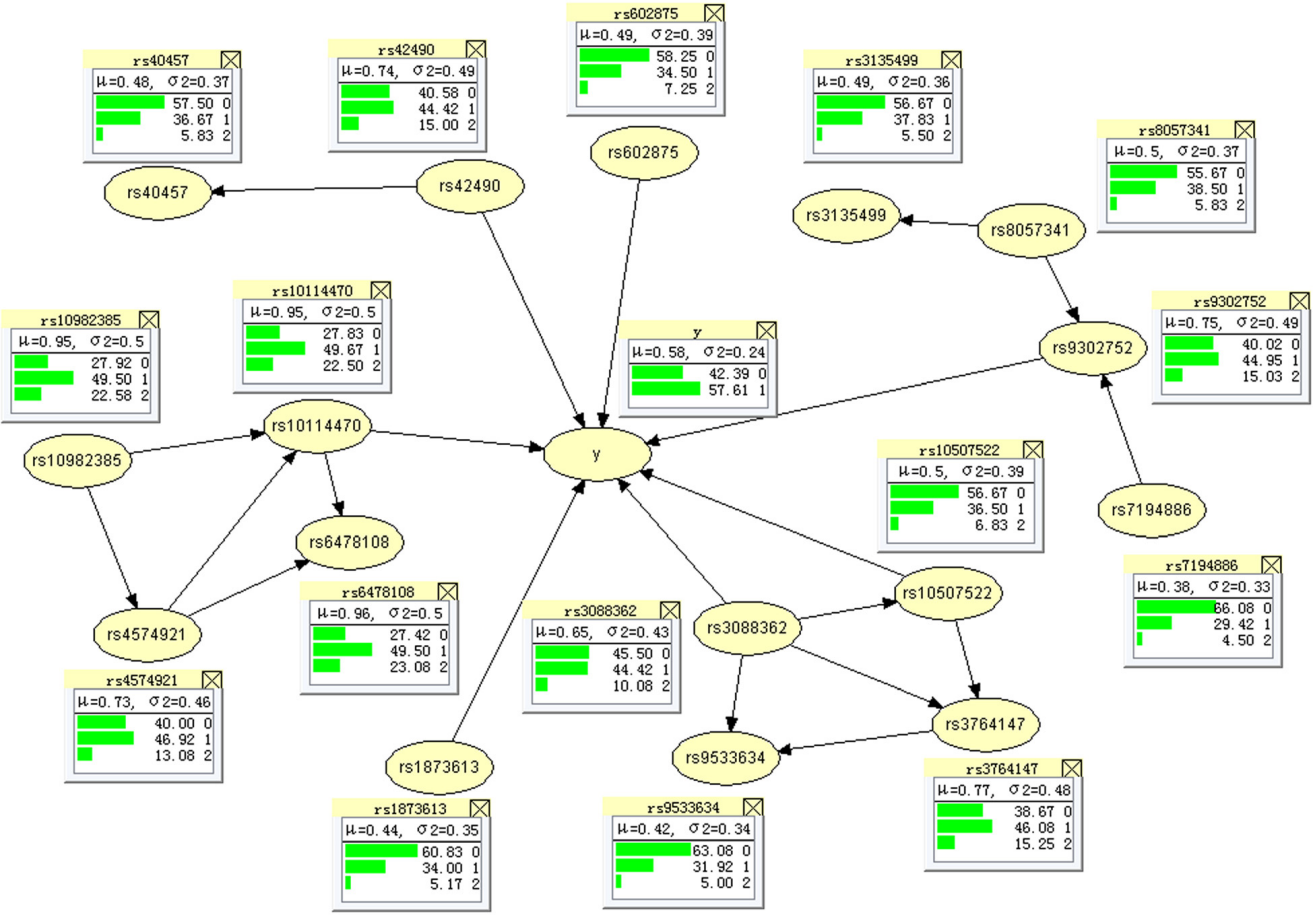
The graphical representation of the Bayesian network in predicting leprosy

Table 2 shows the SNP information and univariate analysis result with Leprosy of the selected 16 SNPs in the model. Seven SNPs entered the multivariate logistic regression model using stepwise approach with results shown in Table 3. Hill climbing method was employed for structure learning and Bayes method for parameter learning using R package bnlearn. Hugin software [31] was used to better visualize the graphical representation of the Bayesian network that is shown in Fig. 4. One hidden layer with four units was used in neural network. Table 4 depicts the AUC and Brier score with 100 repeats of 10-fold cross validation of all the methods. The results show Bayesian network, though just slightly improved, outperforms other two methods, which indicate the network relationships exist in the 16 SNPs. Neural network has inferior performance than the other methods, which may be due to the fact that it is difficult to determine the optimum value for number of hidden layers and nodes.

**Table 2:** SNP information and associations with Leprosy for 16 previously identified SNPs within the Seven Susceptibility Genes

SNP	CHR	Position	Minor allele	Major allele	Gene	MAF	*P* value	OR
rs602875	6	32681607	G	A	HLA-DR-DQ	0.25	3.94E-11	0.54
rs42490	8	90847650	A	G	RIPK2	0.37	5.87E-05	0.71
rs40457	8	90892832	G	A	RIPK2	0.24	7.07E-04	0.72
rs10982385	9	116532838	G	T	TNFSF15	0.47	2.44E-03	1.28
rs4574921	9	116578155	C	T	TNFSF15	0.37	1.74E-04	1.39
rs10114470	9	116587593	C	T	TNFSF15	0.47	4.67E-06	0.68
rs6478108	9	116598524	T	C	TNFSF15	0.48	4.98E-07	0.66
rs1873613	12	38838684	C	T	LRRK2	0.22	3.15E-03	0.75
rs9533634	13	43295815	C	T	CCDC122	0.21	3.97E-04	0.70
rs3088362	13	43331630	A	C	CCDC122	0.32	2.11E-09	1.75
rs3764147	13	43355925	G	A	C13orf31	0.38	2.02E-10	1.74
rs10507522	13	43377000	G	A	C13orf31	0.25	1.97E-08	0.59
rs9302752	16	49276604	C	T	NOD2	0.38	3.09E-12	1.85
rs7194886	16	49282694	T	C	NOD2	0.19	3.43E-07	1.74
rs8057341	16	49295481	G	A	NOD2	0.25	2.13E-03	1.35
rs3135499	16	49323628	C	A	NOD2	0.24	1.81E-03	1.36

**Table 3:** Parameter estimates by multivariate logistic regression

SNP	Estimate	z	*P*	OR
rs602875	-0.636	-6.200	5.63E-10	0.529
rs42490	-0.378	-4.140	3.47E-05	0.685
rs6478108	-0.391	-4.275	1.91E-05	0.677
rs1873613	-0.276	-2.570	0.0102	0.759
rs3088362	0.526	5.154	2.55E-07	1.691
rs10507522	-0.494	-4.735	2.19E-06	0.610
rs9302752	0.665	7.007	2.43E-12	1.945

**Table 4:** The AUC and Brier score of all the methods in predicting leprosy

	AUC	AUC-CV	Brier Score-CV
Bayesian Network	0.7323	0.7199	0.2088
Regression spline	0.7301	0.6986	0.2253
Logistic Regression	0.7441	0.7016	0.2219
Interaction	0.7569	0.6873	0.2304
Neural Network	0.8392	0.6454	0.2597

## Discussion

Several studies demonstrated the importance of investigating a disease from the network perspective. It remains an interesting problem whether the network-based methods have advantageous performance than others, and to what extent do they outperform. The focus of this paper is to bridge this gap and assess their performance in prediction mainly through a series of simulations, with four methods (Bayesian network, neural network, logistic regression and regression splines). We employed the adjusted AUC and Brier score to assess the prediction performance of all the methods. The adjusted AUC are close to 0.5 under null hypothesis when the sample size is larger than 500. It reveals that the discriminatory ability of all methods varies quite slightly with sample size. Four datasets under different assumptions were designed and Bayesian network showed a better performance when the variables are in a network relationship (Fig. 2a) or in a chain structure (Fig. 2c). The regression splines improved the model performance a lot by extracting the nonlinear effect, while the interaction model improved slightly. But they are still inferior to Bayesian network, which indicates that it is not straightforward to capture the whole network information using regression method. For the network structure, we partitioned the effects into additive and non-additive effects to quantify the proportion of the relationships between the input variables and the outcome is non-additive on the logit scale as one reviewer suggested. We have embedded ordinary regression in a larger model including all two-way interactions and calculated the proportion of likelihood ratio chi-square statistics, it showed that 23 % of the effects are due to non-additive effects. The AIC for the additive model and the full model of all the population are 134194.5 and 133034.1 respectively. Particularly, for the special wheel network structure, our simulation results illustrated that the Bayesian network has similar performance of logistic regression model (Fig. 3a), which is strongly consistent with the previous findings [31], same phenomenon has also been found in the case when data was generated using a logistic model (Fig. 3c). Further application on leprosy GWAS show Bayesian network, though just slightly improved, still outperforms other methods, followed by regression splines and logistic regression, and neural network has the worst performance after cross validation. Considering that it seems to be unreasonable to predict leprosy using the non-risk SNPs, we thus have chosen the specific 16 risk SNPs which have been identified and validated from the GWAS of leprosy.

Logistic regression models are well suited to be used when some assumptions is satisfied (Fig. 3c), while they work inferior when the assumptions are violated and cannot capture the nonlinear and unknown relationships often existed in the variables. It would be of great value to add penalized MLE to the comparators to make the comparison with logistic regression more informative, which remains a goal of our future work. Neural networks can reflect the complex relationships between the predictor variables and the outcome by the hidden nodes in the hidden layer. However, as a weighted average of logit functions with the weights themselves estimated, it does not jump out from the scope of regression yet. Moreover, the network structure must be pre-specified and no gold standard can be adopted to determine the optimum value for number of hidden layers and nodes. Bayesian networks capture the complex relationship well between a larger number of predictors with their interactions without statistical assumptions, when the disease is caused through pathways or networks, and the usefulness of Bayesian networks for predicting is clearly recognized through simulation. Even when the dataset were generated from regression model, the Bayesian network techniques had a considerate performance (Fig. 3c). Actually, the Bayesian network is confirmed theoretically to be equivalent to a logistic regression problem under a simple graph-theoretic condition (e.g. wheel network in our simulation) [31, 32]. One major drawback of Bayesian network is that its performance can be heavily influenced by the network structure, which sometimes may not capture the real population structure information, though many algorithms have been provided for network structure learning.

These comparisons are dependent on the character of a particular data set, and one cannot conclude whether one method will be superior to the others in a given data set without dissecting the data structure. Overall, regression-based methods are recommended for well-designed research projects with a small amount of variables where researchers can understand the potential predictors and possible interactions, since it is easier to be implemented and to be accepted by clinical researchers. For the dataset with complex relationships, especially for commonly accepted network-centric perspective for complex disease, network-based methods such as Bayesian network are more appropriate to act as an exploratory tool. These methods can extract the patterns and relationships in data without constraining the predictors, and achieve a high performance in discrimination.

## Conclusion

Although regression-based methods are still popular and widely used, network-based approaches should be paid more attention, since it captures the complex relationship between variables.

## Additional file

Additional file 1: Relevant tables for the comparison of Brier score. (DOCX 18 kb)

## Abbreviations

AUC: The area under the receiver-operating characteristic curve
AUC-CV: The AUC using 10-fold cross validation
BN: Bayesian network
CV: Cross validation
GWAS: Genome-wide association study
NN: Neural network
RS: Regression splines
