# Supplementary material for: Network or regression-based methods for disease discrimination: a comparison study
Source: BMC Med Res Methodol. 2016 Aug 18;16:100. doi: 10.1186/s12874-016-0207-2 (PMC4991108; doi:10.1186/s12874-016-0207-2)
Supplement: Additional file 1: — Relevant tables for the comparison of Brier score. (DOCX 18 kb) [file 12874_2016_207_MOESM1_ESM.docx]

Table S1 Brier score of the three methods for chain network

| **Method** | **Brier score with 10-fold CV** | | | | | |
| --- | --- | --- | --- | --- | --- | --- |
|  | 30 | 50 | 100 | 200 | 500 | 1000 |
| Bayesian network | 0.29306 | 0.28279 | 0.26194 | 0.24582 | 0.22982 | 0.22410 |
| Neural network | 0.29458 | 0.27934 | 0.26983 | 0.23521 | 0.23357 | 0.23213 |
| Logistic Regression | 0.29474 | 0.27564 | 0.27184 | 0.25219 | 0.24858 | 0.24681 |

Table S2 Brier score of the three methods for wheel network

| **Method** | **Brier score with 10-fold CV** | | | | | |
| --- | --- | --- | --- | --- | --- | --- |
|  | 30 | 50 | 100 | 200 | 500 | 1000 |
| Bayesian network | 0.26710 | 0.24924 | 0.23641 | 0.22977 | 0.22625 | 0.22393 |
| Neural network | 0.27181 | 0.25023 | 0.24076 | 0.23152 | 0.22936 | 0.22476 |
| Logistic Regression | 0.29826 | 0.26508 | 0.24515 | 0.23491 | 0.23028 | 0.22469 |

Table S3 Brier score of the three methods for data generated from logistic regression

| **Method** | **Brier score with 10-fold CV** | | | | | |
| --- | --- | --- | --- | --- | --- | --- |
|  | 30 | 50 | 100 | 200 | 500 | 1000 |
| Bayesian network | 0.22246 | 0.20599 | 0.19128 | 0.18146 | 0.17357 | 0.17067 |
| Neural network | 0.23066 | 0.20986 | 0.19213 | 0.18053 | 0.17205 | 0.17088 |
| Logistic Regression | 0.20875 | 0.19018 | 0.18884 | 0.17840 | 0.17051 | 0.16945 |
